# Vascular malformations: An overview of their molecular pathways, detection of mutational profiles and subsequent targets for drug therapy

**DOI:** 10.3389/fneur.2023.1099328

**Published:** 2023-02-10

**Authors:** Ann Mansur, Ivan Radovanovic

**Affiliations:** ^1^Division of Neurosurgery, Department of Surgery, Faculty of Medicine, University of Toronto, Toronto, ON, Canada; ^2^Department of Laboratory Medicine and Pathobiology, School of Graduate Studies, University of Toronto, Toronto, ON, Canada; ^3^Division of Neurosurgery, Department of Surgery, Toronto Western Hospital, University Health Network, Toronto, ON, Canada; ^4^Krembil Brain Institute, University Health Network, Toronto, ON, Canada

**Keywords:** vascular malformation (VMs), targeted therapy, precision medicine, liquid biopsy, signaling pathway

## Abstract

Vascular malformations are anomalies in vascular development that portend a significant risk of hemorrhage, morbidity and mortality. Conventional treatments with surgery, radiosurgery and/or endovascular approaches are often insufficient for cure, thereby presenting an ongoing challenge for physicians and their patients. In the last two decades, we have learned that each type of vascular malformation harbors inherited germline and somatic mutations in two well-known cellular pathways that are also implicated in cancer biology: the PI3K/AKT/mTOR and RAS/RAF/MEK pathways. This knowledge has led to recent efforts in: (1) identifying reliable mechanisms to detect a patient's mutational burden in a minimally-invasive manner, and then (2) understand how cancer drugs that target these mutations can be repurposed for vascular malformation care. The idea of precision medicine for vascular pathologies is growing in potential and will be critical in expanding the clinician's therapeutic armamentarium.

## Introduction

**Figure 1:**
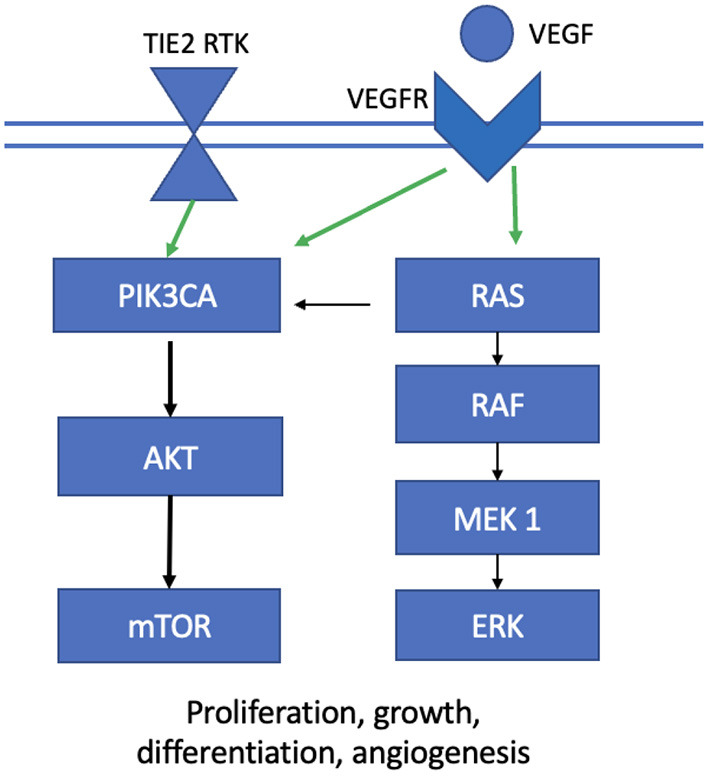
Two major cellular signaling pathways implicated in the development of vascular malformations. TIE2 RTK, *TEK* gene receptor tyrosine kinase; VEGF, vascular endothelial growth factor; VEGFR, vascular endothelial growth factor receptor.

Vascular malformations (VMs) are inborn errors of vascular development that result in abnormally formed vessels. They are classified into slow- and fast-flow malformations based on the absence or presence of an arterial component, respectively. They include venous, lymphatic, arterial, capillary and mixed malformations, alongside those associated with other anomalies (1). While majority of VMs are present at birth, they can sometimes form *de novo* in post-natal development. Of those present at birth, 5% are caused by inherited loss-of-function (LOF) germline mutations with a somatic second hit, while the remaining 95% are sporadic in nature, meaning that they occur due to gain-of-function (GOF) mutations occurring after conception in clusters of non-gametal cells (2). These mutations occur in one of two major cellular signaling pathways: the PI3KCA-AKT-mTOR and the RAS-RAF-MEK-ERK pathways, which govern angiogenesis, cell growth and proliferation, motility and apoptosis (Figure 1). Both pathways are implicated in various cancers and through their assessment in these populations, targeted therapeutics were developed that address various aspects of these intricate cascades. These systemic agents are being introduced in the management of vascular malformations, albeit at a largely experimental level. This review summarizes the literature to date on the molecular pathophysiology of VMs and novel methods to detect their mutational burden, alongside an update on their potential targeted therapies.

## Slow-flow malformations

Slow-flow malformations include venous malformations (VeMs), lymphatic malformations (LMs), and mixed vascular malformations, alongside overgrowth syndromes. These malformations are characterized by overactivation of phosphoinositide 3-kinase (PI3K) through mutations in the PI3K/AKT/mTOR signaling pathway (3–5). In the normal state, ligand binding results in phosphorylation of phosphatidylinositol-4,5-bisphosphate to phosphatidylinositol-3,4,5-triphosphate through PI3K, which then recruits the downstream target AKT (6). Phosphate and tensin homolog (PTEN) normally inhibits P13K, thereby downregulating this signal cascade (Figure 1).

VeMs are soft and compressible blue lesions that typically occur in the skin or mucosal membranes. Most are sporadic and are caused by somatic GOF mutations in the *TEK* gene, which encodes the endothelial receptor tyrosine kinase TIE2 (7). When angiopoietin 1 (ANPT1) binds TIE2 in endothelial cells, it then activates the canonical PI3K-AKT-mTOR pathway leading to endothelial cell proliferation (2, 8). Somatic *TIE2* mutations occur in unifocal and multifocal VMs and blue rubber bled nevus syndrome (7–9). Those with inherited cutaneomucosal VeMs have autosomal dominant *TIE2* mutations and together with a somatic second hit mutation in *TEK*, these patients then demonstrate their multifocal lesion phenotype (10).

**Figure 2:**
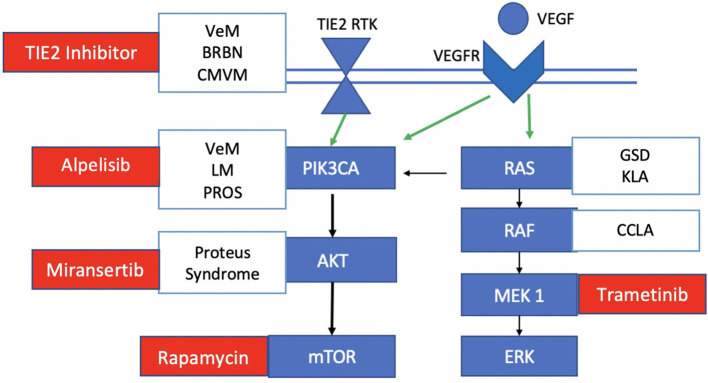
Mutations and targeted therapies for slow-flow malformations. TIE2 RTK, *TEK* gene receptor tyrosine kinase; VEGF, vascular endothelial growth factor; VEGFR, vascular endothelial growth factor receptor. Somatic TIE2 mutations are observed in unifocal and multifocal venous malformations (VeMs), blue rubber bled nevus syndrome (BRBN) and cutaneomucosal venous malformations. Somatic PIK3CA mutations are seen in venous malformations, lymphatic malformations and PROS. Some central lymphatic anomalies such as Gorham Stout Disease (GSD) and kaposiform lymphangiomatosis (KLA) harbor RAS mutations while patients with central conducting lymphatic anomalies (CCLA) have ARAF mutations. Currently investigated targeted therapies for each component of the pathway in slow-flow malformations are shown in red.

A smaller subset of VeMs (20%) harbor somatic mutations in the *PIK3CA* gene, which encodes the alpha subunit of the downstream effector PI3K (11, 12). In endothelial cells, the alpha subunit is activated by the VEGF and TIE receptors to eventually phosphorylate AKT, especially its AKT1 isoform (13, 14) (Figure 2). Similar to *TIE2* mutations, a GOF mutation in *PIK3CA* thereby leads to overactivation of AKT (15). These mutations are also commonly found in *PIK3CA*-related overgrowth syndromes (PROS) such as Klippel-Trenaunay syndrome (KTS), megalencephaly-capillary malformation syndrome (MCAP), and congenital lipomatous overgrowth with vascular anomalies, epidermal nevi and scoliosis syndrome (CLOVES) (16, 17) (Figure 2). These syndromic forms of LM have more wide-spread *PIK3CA* mutations in non-hot spot loci (18). Proteus syndrome, another overgrowth syndrome, harbors somatic mutations in the downstream effector *AKT* (19).

LMs (microcystic and macrocystic) and complex lymphatic anomalies (CLAs) are localized or multifocal lesions of the lymphatic vasculature, respectively (17). CLAs include Gorham-Stout Disease (GSD), generalized lymphatic anomalies, central conducting lymphatic anomalies (CCLA), and kaposiform lymphangiomatosis (KLA) (20). In normal lymphatic vascular development, differentiation is initiated by the *PROX1* gene and maintained by VEGF signaling (21). Postnatally, vessel morphology is matured by angiopoietin-2 (ANGPT2) via the TIE2 receptor that is involved in PI3K-AKT-mTOR signaling; valves are developed under the regulation of the EPHB4 receptor tyrosine kinase, which recruits RASA1 to normally inhibit the RAS-MAPK-ERK signaling pathway (18) (Figure 2). Hence, lymphatic development relies on transduction of both the PI3K-AKT-mTOR (for sprouting and maturation) and RAS-MAPK-ERK (for growth and proliferation) pathways. Like VeMs, the majority of cystic LMs harbor the same types of *PIK3CA* hot-spot mutations which cause increased PI3K activity as well as increased phosphorylation of downstream AKT (15, 22). More rarely, some patients with CLA have mutations in the RAS-MAPK/ERK pathway including *KRAS, NRAS* and *EPHB4* (23–26). In addition to these two cellular pathways, the LM growth is supported by paracrine secretion of VEGF, likely mediated by immune cells (27).

If left untreated, slow-flow malformations harbor significant morbidity, commonly presenting with lesions, coagulopathy, thrombosis, pain/migraine, and location-specific functional limitations that worsen through puberty. Lesions of the central nervous system (CNS) include: (1) developmental venous anomalies that are benign but can present with thrombosis leading to hemorrhage or infarction with an incidence of 0.22–0.68% per year, and even higher if associated with cerebral cavernous malformations; (2) sinus peri-cranii which can result in headaches, tinnitus or issues with cosmesis that cause impairments in quality of life, and (3) cavernous malformations that occur with an incidence of 0.1–0.8% and present with seizures in up to 50% of patients, hemorrhage in 25% and focal neurological deficits in 5–10% of patients (9, 17, 28). Generally, CNS lesions can have significant morbidity if a patient is plagued with intractable seizures, headaches, tinnitus, cranial nerve dysfunction, and even heart failure in newborns. Some slow-flow malformations are life-threatening due to extension into vital tissues such as the airway, due to cardiac failure, or due to severe impairments in coagulopathy and uncontrolled bleeding; mortality rates for slow-flow malformations if left untreated range from 0.1 to 20%, with VeMs and deep seated cerebral cavernous malformations, respectively (28).

Standard treatments of these slow-flow malformations include: (1) supportive management with compression, analgesia and correction of coagulopathy; (2) medical management of seizures and heart failure; (3) surgical resection; (4) sclerotherapy and/or laser therapy for symptom relief and cosmesis; and (5) endovascular treatment for specific slow-flow malformations such as accessory sinus pericrania. Laser ablation is typically used for superficial dermal or mucosal lesions, and surgery is often limited to focal lesions in easily accessible locations to ensure a complete resection, which if cured can significantly reduce the morbidity and mortality rate. Sclerotherapy with either doxycycline, bleomycin or 3% sodium tetradecyl sulfate is frequently used but often repeated treatments are needed, yet insufficient for cure; sclerotherapy is complicated by skin necrosis, pain, blistering and peripheral neuropathy in up to 10% of patients, and less commonly by venous thromboembolism/pulmonary embolism, pulmonary fibrosis and cardiopulmonary failure (28, 29). Treatment of patients with CLAs and syndromic VeMs is particularly challenging given the extensive tissue involvement and deep location of vascular lesions; an approach with systemic agents would be useful in targeting the molecular biology of the disease, and particularly ideal for these complex syndromes. To date, there are several systemic agents being investigated for slow-flow malformations that target different parts of the PI3K-AKT-mTOR and RAS-MAPK-ERK signaling pathways (Figure 2).

### PI3K-AKT-mTOR pathway inhibitors

#### MTOR inhibitor

Rapamycin (also known as sirolimus) a is a commonly used drug in clinical practice for its immunosuppressive and antiangiogenic properties. It works by disrupting mTORC1, thereby preventing it from phosphorylating downstream proteins S6Rp and 4E-BP1, which are critical for cellular differentiation, proliferation and motility (30). Its relevance to VMs was only recently discovered. In a mouse model of VMs with injected TIE2^L914F^-mutated endothelial cells, mice that were injected with rapamycin-pretreated cells had smaller and more poorly developed vasculature than those who did not get the rapamycin-pretreated or TIE2 inhibitor-pretreated cells (31). Established VMs in both the mice and murine models treated with rapamycin had delayed growth and reduced VM volume compared to vehicle and TIE2 inhibitor-treated groups (31).

Rapamycin was assessed in a variety of retrospective studies for both pediatric and adult patients with severe slow-flow malformations including syndromic VMs and CLAs (32–35). The starting dose was 2mg daily for adults and 0.8 mg/m^2^ twice daily for children, with target trough levels of 10–15ng/mL. At this dose, rapamycin was well tolerated with mostly minor yet frequent adverse events necessitating conservative management. Twelve patients had grades 1–2 mucositis, four had opportunistic infections, one had a headache and one had hypertension; four of these patients required a dose reduction and only one patient discontinued the treatment due to severe mucositis. On laboratory testing, 10 patients had hyperlipidemia, four had elevated liver enzymes and one patient had lymphopenia. Patient symptoms, including pain, hemodynamic instability, and functional status improved in over 80% of patients within 3 months; no patient had complete cure with rapamycin treatment.

A pilot prospective trial on rapamycin in six adult patients with palliative slow-flow VMs showed clinical efficacy with significant reduction in pain, over 50% improvement in quality of life, decreased D-dimer levels, and improved signs of coagulopathy, all of which occurred in the first 3 months (31). Magnetic resonance imaging follow-up scans showed an average of 20% reduction in the size of the lesions and in some, complete disappearance of the lesion (31). The follow up phase IIB study included 19 patients with severe slow-flow VMs who all benefited from an improvement in quality of life within the first 3 months of daily rapamycin treatment (36).

The clinical efficacy of Rapamycin in patients with PROS is less evident (37, 38). One study on 39 patients with PROS trialed low-dose rapamycin (2–6 ng/mL) and found that while there was an observable effect on lesion overgrowth, it did not translate into functional improvements and quality of life (38). It is unclear if this is due to the lower dose target.

Finally, the VASE trial is the largest Phase III multicentre trial currently underway to evaluate the efficacy of rapamycin in pediatric and adult patients with slow-flow VMs refractory to standard treatment (EudraCT2015-001703-32; https://www.clinicaltrials.gov; NCT02638389). Rapamycin is administered over a two-year period before being stopped; patients can resume the treatment if symptoms resurge. While they aim to enroll 250 patients, the preliminary analysis on the first 101 patients with at least six months follow up showed that 87% had improvements in their pain and functional outcome. Thirty-six patients were able to stop the medication after 2 years, of which half needed to restart the medication due to eventual symptom resurgence. Queisser et al. recently reviewed the data on rapamycin's clinical efficacy in severe slow-flow VMs; in combining the data from the Phase II and VASE Phase III trials (*N* = 122), they report an 85% toxicity rate, mostly constituting mild adverse events that were managed conservatively (9). The most common side effects included fatigue, nausea, headache and cutaneous rash. In 18% of patients, a dose reduction or temporary arrest was sufficient in alleviating these mild adverse events whereas a definitive arrest was needed in 10% of patients. For patients undergoing surgical resection after therapy with sirolimus, the preliminary experience in patients with VMs is that it should perhaps be maintained during surgical management in order to facilitate wound healing through decreased lymphatic leakage, which is contrary to the experience in oncological population (39). More surgical studies are needed to validate the drug's effect on wound healing in this particular population.

While tolerance of rapamycin is good with mild-to-moderate and manageable side effects, optimizing dose requirements is clearly still important in ameliorating the drug's safety profile. In studying rapamycin's pharmacokinetics, strategies in altering dose targets and routes of administration have recently been explored, in addition to adding prophylactic agents to target the more common side effects. In one approach, Harbers et al. retrospectively analyzed the effect of a lower drug target level in 12 patients with therapy-resistant low-flow VMs; they found that in doing so, they achieved similar clinically efficacy with reduced adverse events using low rapamycin target levels of 4–10 ng/ml (40). A lower drug target for rapamycin in a study on PROS patients, however, did not reach its intended safety effect (38). It's unclear if this is related to rapamycin's effect in PROS patients specifically or it is a mere reflection of patient heterogeneity. A body-surface area dosing approach often unnecessarily exposes tissues outside of the lesion target to the drug; instead, we know that in the pediatric population, the cytochrome P450 metabolic activity evolves through development and with this knowledge, an age-appropriate and pharmacokinetic based rapamycin dosing regimen was proposed (41, 42).

A topical route might also help to alleviate some of these toxicities; topical sirolimus at 1% concentration was recently assessed in a systemic review on 23 patients with cutaneous vascular anomaly manifestations and found to be effective in improving cutaneous lesion appearance in 86% of patients and improving lymphatic blebbing in 90% of patients over an average length of treatment of 10.2 months (43). There were no severe adverse events although one patient electively stopped treatment due to pruritis (43). A multicentre randomized controlled trial is planned to assess topical sirolimus for patients with lingual microcystic LMs, where other treatment options are quite limited (www.clinicaltrials.gov; NCT04128722).

Lastly, prophylactic agents for common or severe reactions were used in some studies, including the use of mouth washes and topical agents for stomatitis or antibiotics for opportunistic pneumonia in the solid organ transplant population; however, there is currently insufficient evidence to support guidelines for prophylactic measures for patients with VMs and these practices remain at the physician's discretion.

#### PI3K inhibitor

Alpelisib (BTL719) is an oral specific allosteric inhibitor of PI3K by the company Novartis that selectively inhibits the p110alpha subunit (44). It acts by inhibiting the phosphorylation of downstream target AKT. Alpelisib was initially studied in patients with *PIK3CA*-altered oncological diagnoses, including Phase I trials in advanced solid tumors (45–47) and more recently, an ongoing Phase III trial on alpelisib with fulvestrant for patients with hormone receptor-positive advanced breast cancer (SOLAR-1 trial) (48). Evidence from the oncology literature demonstrated significant improvements in progression-free survival when combined with other chemotherapeutic agents with mostly mild side-effects including hyperglycemia, gastrointestinal symptoms, cutaneous irritations, fatigue and mucositis (48). Severe reactions including diabetic ketoacidosis, diarrhea, hypertension, hypersensitivity reactions, osteonecrosis of the jaw, and severe cutaneous adverse events have all been reported, and occur in up to 35% of patients in the SOLAR-1 trial (48).

Alpelisib was then tested in a preclinical study on PROS/CLOVES mutant mice with established lesions. They found that like rapamycin, alpelisib improved vascular morphology and reduced lesion size but was superior to rapamycin in restoring organ dysfunction (49). Isolated case reports in infants with severe PROS demonstrated similar clinical improvements and reductions in lesion volume with no adverse events (50). A larger clinical prospective study on 19 patients with PROS trialed alpelisib at 250 mg daily for adults and 50 mg daily for children; they found that the drug was well tolerated with grade one hyperglycemia in two patients and mucositis in three patients. Importantly, all patients experienced significant clinical improvement in their symptoms alongside reduction of lesion volume by 30% within 6 months of daily use (49). Six of these patients had trialed rapamycin without success, which corroborates the role of rapamycin in treating actively growing lesions (38) while alpelisib can reverse existing lesions in this population.

Alpelisib's clinical efficacy in the PROS population was later supported by the findings of the EPIK-PP1 (NCT04285723) study, which is a retrospective chart review on patients with PROS above the age of 2 with severe or life-threatening disease. These patients had confirmed mutation in the *PIK3CA* gene and took at least one dose of alpelisib (adult 250 mg/d; pediatric: 50 mg/d) for >24 weeks before March 2020 (4). Their primary outcome was radiological response with at least 20% reduction in sum of the target lesion volume in up to three lesions confirmed on repeat imaging. Secondary objectives included safety and clinical efficacy with change in patient symptomatology. Of the 57 patients (39 pediatric, 18 adult) included in this study, 32 patients had complete follow up data for primary endpoint analysis. Twelve patients (37.5%) (95% CI: 21.1–56.3%) were radiological responders by Week 24 and 60% of these patients had sustained response for at least 1 year. Improvements in symptomatology were reported in 90.9% of patients for pain, 78.9% for VM appearance, 76.2% for fatigue, 69.0% for limb asymmetry, and 55.2% for disseminated intravascular coagulation. Treatment-related adverse events were mild and occurred in 38.6% of patients, of which the most common were hyperglycemia, stomatitis and self-limiting mouth ulcers. Based on such robust evidence of its safety and efficacy in this population, Alpelisib (Vijoice R) (51) was just recently approved by the American Federal Drug Administration under a Managed Access Program for patients with severe PROS requiring systemic treatment (Novartis: Vijoice; www.clinicaltrials.gov; NCT NCT04085653). A phase 2 upfront 16-week randomized control trial on the pharmacokinetics, safety and efficacy of alpelisib for pediatric and adult PROS patients is underway with the anticipated study completion date being in December 2029 (www.clinicaltrials.gov, NCT NCT04589650).

There have only been preclinical studies and small case series on oral and topical alpelisib for other isolated VMs, demonstrating restored vascular morphology, reduced lesion size, improved fibronectin levels and improvements in symptoms such as pain and inflammatory flares (11, 12, 52, 53). In one study, these effects were similar to mTOR inhibition (12), whereas in the other it was significantly better than rapamycin (11). It's clinical application to patients with other slow-flow malformations remains unclear at this time and requires further large prospective trials to confirm its safety and efficacy in distinct VM populations (54).

#### AKT inhibitor

Miransertib (ARQ 092) is an oral selective AKT inhibitor with higher specificity for the AKT1 isoform; it works by inhibiting the membrane-bound active form of ACT and preventing the conversion of its inactive form to its active state (55). It was initially investigated in oncological models (55, 56) before being assessed in preclinical models of *PIK3CA*-mutant endothelial cells, demonstrating regression of existing proliferating VM lesions (5). Its clinical efficacy has most recently been explored only in a handful of case reports and series of patients with PROS/CLOVES (5, 57, 58). In 6 patients with PROS, miransertib had greater antiproliferative activity as measured by fibroblasts compared to mTOR inhibitors (57). In a preclinical study on human derived endothelial cells from *PIK3CA* and *TEK/TIE2* driven VMs, miransertib impacted the viability of the endothelial cells at even low concentrations and helped to restore the wild-type endothelial cell phenotype (5). In a case report on a 16-year-old female patient with CLOVES that progressed on rapamycin, miransertib at 30 mg daily dose resulted in 15% reduction in fatty overgrowth volume over 28 months and improvements in respiratory function with mild hyperlipidemia as a side effect; however, treatment was discontinued due to lack of sustained response and poor patient adherence after 28 months (58). There is currently an ongoing phase I/II trial on Miransertib for patients with PIK3CA-related overgrowth diseases and Proteus syndrome (MOSAIC-study: www.clinicaltrials.gov; unique identifier NCT03094832).

### RAS-MAPK-ERK pathway inhibitors

#### MEK inhibitor

Trametinib is an allosteric MEK1/MEK2 inhibitor from the company Novartis that is Health Canada and Food and Drug Administration approved for certain *KRAS* pathway-driven cancers, including metastatic melanoma and non-small cell lung cancer. It however was recently applied to both a transgenic zedbrafish model of CCLA and a single patient with advanced CCLA with *ARAF* mutation (59). They found that MEK inhibition restored the endothelial cell monolayer and adherens junctions and rescued duct morphology without changes in cell proliferation in the preclinical model. In the 12-year-old patient with CCLA, they observed an improvement in pulmonary function with reduced lymphatic fluid retention and oxygen requirements in the first 3–6 months of treatment that was sustained after 1 year (59). Similarly, in both a transgenic mouse model of GSD and patient harboring *KRAS* G12V somatic mutation, Trametinib regressed the lymphatic valves, improved chylothorax and restored lymphatic flow (60). Finally, in another patient with KLA and *NRAS* mutation, trametinib was well tolerated and resulted in improved clinical symptoms (61) (Figure 2).

Overall, there is good evidence for the clinical efficacy of oral rapamycin for patients with severe VeMs, LMs and mixed VMs (32, 36, 37, 40, 62), and promising future in the application of direct PIK3CA and AKT inhibitors for patients with PROS. MEK inhibitors may play a role for RAS-RAF-ERK pathway driven lymphatic anomalies and possibly complimentary to PI3K-mTOR pathway targeted therapies as a combinatorial approach for inhibiting angiogenesis and proliferation in these patients. Further work is needed on determining pharmacokinetically-driven drug regimens for each agent in specific VM subpopulations, and then trialing a combinatorial approach to better address tolerance and resistance in chronic therapy.

#### High-flow malformations

**Figure 3:**
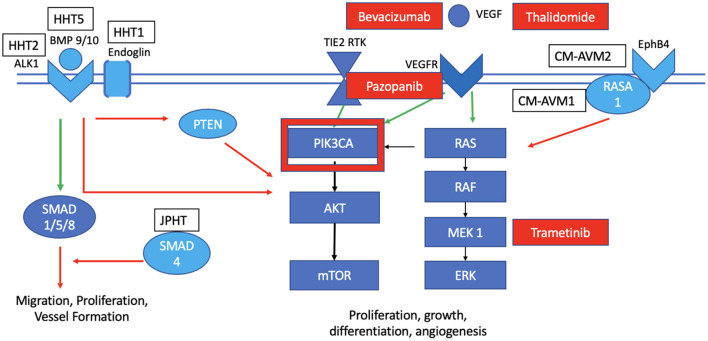
Mutations and targeted therapies for slow-flow malformations. HHT1 is known to have multiple affected loci in the BMP signaling pathway that generally act to inhibit the PIK3CA pathway in the wild-type state. CM-AVM 1 is characterized by a LOF mutation in RASA1, while CM-AVM2 has the mutation in the EphB4; both activate the KRAS pathway in the wild-type state. Targeted therapies for HHT include bevacizumab, pazopanib and thalidomide. There is a potential to also explore PI3KCA with Alpelsib for HHT. Targeted therapies for CM-AVM include Trametinib, while those for sporadic AVMs include bevacizumab, thalidomide, and Trametinib.

Our initial insight into the molecular biology of high-flow VMs was based on an assessment of germline inherited mutations in familial vascular syndromes. These mutations are inherited in an autosomal dominant fashion with a LOF mutation followed by a second somatic hit. To date, the most well-known familial AVM disorders include hereditary hemorrhagic telangiectasia (HHT) and capillary malformation- arteriovenous malformation syndrome (CM-AVM) (Figure 3).

HHT (Osler-Weber-Rendu syndrome) manifests as multiple cutaneous telangiectasias, recurrent epistaxis or gastrointestinal bleeding, and AVMs in various internal organs, including the lungs, liver and central nervous system. In HHT, there are five loci implicated in the development of this disease, most of which involve the bone morphogenetic protein (BMP) signaling pathway and are upregulated alongside VEGF signaling. Both HHT1 and HHT2 are caused by heterozygous LOF mutations in *endoglin* (*ENG*) and *activin receptor-like kinase 1* (*ALK1*), respectively. HHT syndrome (also known as juvenile polyposis) is linked with LOF mutations in genes that encode SMAD (mothers against decapentaplegic homolog). Other loci were found implicated on chromosome 5q31.3-32 (HHT3) and 7p14 (HHT4). *ALK1* and *ENG* are expressed on endothelial cells. *ENG* induces the BMP/ALK1 signaling cascade by receptor phosphorylation and activation of various SMAD transcription factors that typically suppress endothelial cell migration and proliferation (Figure 3). Hence, LOF mutation in these genes disinhibits these activities, causing enhanced aberrant vessel formation. Similarly, experiments with knockout or blockade of ALK1 and BMP9/10 ligand demonstrate inhibition of PTEN and an associated increased activation of AKT.

Systemic therapies for HHT have largely focused on inhibiting VEGF directly or indirectly. Bevacizumab (Avastin) is a widely used recombinant monoclonal antibody that inhibits VEGF and was studied in several clinical trials in patients with HHT (39, 63, 64). They observed improvements in liver lesions, telangiectasis and bleeding (especially refractory gastrointestinal bleeding) (64) with no serious adverse events. Intramucosal injections were superior to intravenous or intranasal administrations in this population (39, 64). There is now a randomized phase III clinical trial underway studying the safety and efficacy of bevacizumab for patients with HHT (NCT 03227263). Recently, indirect inhibitors of VEGF signaling such as pazopanib (inhibits various tyrosine kinase receptors such as VEGFR2) (65) and thalidomides were experimented in a small number of patients with HHT with some early success in reducing bleeding (64, 66). There are now ongoing trials planned in North America to study the clinical utility of these anti-angiogenic drugs for severe cases of HHT (NCT03850730, NCT03850964). Finally, common downstream effectors such as PI3K may be future candidates for targeted drug trials since they showed promise in reducing the levels of the BMP9/10 antibodies in the mouse model (67, 68) (Figure 3).

Patients with CM-AVM have multifocal maroon-colored lesions and sometimes high flow lesions including AVMs. CM-AVM1 is caused primarily by *RASA1* mutations. *RASA1* encodes the RAS p21 protein activator 1 (p120RasGAP) that when recruited, it inhibits the RAS/MAPK/ERK signaling pathway; hence, LOF mutations in *RASA1* cause abnormal activation of this pathway with increased cellular proliferation, growth, differentiation and motility. CM-AVM2 is caused by LOF mutations in the transmembrane receptor *EPHB4* that is expressed on venous cells, which interacts with p120RasGAP to inhibit the RAS/MAPK/ERK signaling pathway. Once again, mutated *EPHB4* then leads to constitutive activation of this pathway (69). Given the upregulation of the RAS/MAPK/ERK pathway in these diseases, MEK inhibition can perhaps play a role in targeting this aberrant pathology (Figure 3). A case report was published just this year on the successful management of a 16-year-old female patient with CM-AVM and cardiac compromise (70). Further studies are needed to elucidate the role of pathway-targeted treatments such as Trametinib in the care of CM-AVM patients.

Most patients with fast-flow lesions have sporadic AVMs that are not caused by these inherited LOF mutations. Without any intervention, the hemorrhage rate for AVMs is around 2% and higher (around 4–10%) if there is a previous rupture or an angiographic weak point (71). These lesions are typically treated with surgery, radiosurgery, embolization or a combination of these approaches with a risk of intervention being variable depending on the anatomy and location of the lesion; however, many lesions are either too complex to achieve a cure with the most aggressive of these approaches or they are located in areas that are difficult to access. Hence, systemic therapies can play an important role in this debilitating disease. Early studies on targeted therapies for AVMs focused on decreasing the angiogenic upstream stimulus or the resultant inflammation that weakens the vessel wall (Figure 3). In an open label pilot study on the safety of tetracycline derivatives in 12 adult patients with inoperable AVMs and 14 patients with giant cerebral aneurysms, Frenzel et al. found that a third of these patients had dose-limiting intolerance (72). Bevacizumab was also trialed in this population to reduce the angiogenic activity but while there was a reduction in serum VEGF levels with no serious drug toxicity, this did not translate into clinical improvements in the two patients with AVMs (73). The most promising systemic agent targeting upstream actors in the angiogenic signaling pathway for AVM care is thalidomide. This drug was initially used as a sedative for pregnant women decades ago and was stopped for its teratogenic side effects. In this pursuit, we also learned that it has antiangiogenic properties by inhibiting cytokines such as VEGF, affecting cell migration and adhesion, and modulating the inflammatory response through inhibition of tumor-necrosis factor alpha and nitric oxide (74). Given its anti-angiogenic and anti-inflammatory potential, it was tested in patients with age-related macular degeneration, HHT and explored in an AVM mouse model (39). Boon et al. have since highlighted a prospective observational case series of 18 adult patients with severe extracranial AVMs and functional impairment who were treated with thalidomide (66). They found that all patients experienced a reduction in pain and improvement in both bleeding and ulceration. Two patients (11.1%) had concomitant reduction in vascularity on follow-up angiography. Of the 12 patients who stopped thalidomide treatment due to clinical improvement, 8 remained stable and 4 had lesion recurrence within the first year. The first 5 patients were administered the drug at initial dose of 50 mg per day which was escalated to 200 mg daily within the first 2 weeks. At this dose regimen, 80% of these patients had grade 3 complications including asthenia, erythroderma and cerebral infarct (the latter was unclear if caused by thalidomide). The remaining patients were trialed on the lower dose regimen of 50 mg daily with no significant effect on the drug's efficacy but an observable reduction in the rate of toxicity (66). Further prospective trials on thalidomide in patients with severe AVMs are needed to determine its safety and efficacy in clinical practice.

These studies all predate a seminal discovery in 2017 that the overwhelming majority of sporadic brain AVMs harbor activating *KRAS* mutations in nidal endothelial cells (75) (Figure 3) and sporadic extracranial AVMs have mutations in *MAP2K1* (76). *In vitro* and immunohistochemistry experiments demonstrated that mutant KRAS expression increased downstream ERK phosphorylation, increased expression of angiogenic signaling, and enhanced the cell's migratory behavior. With deeper genomic sequencing depths, *KRAS* mutant prevalence increases to almost 90% of brain and spine AVMs and is sufficient to induce AVMs in preclinical models (77, 78). Mosaic variants in components of the *KRAS* pathway (*KRAS, NRAS, BRAF* and *MAP2K1*) were discovered as activating drivers in the development of extracranial AVMs as well (79). Their aberrant features were reversed through *in vitro* and preclinical *in vivo* experiments with MEK inhibition. Hence, there is a growing body of knowledge that *KRAS* mutations drive sporadic AVM development (80); this pathway then serves as a logical target for therapeutic drug discovery with MEK inhibition.

To date, isolated case reports illustrated the application of Trametinib to pediatric patients with severe extra-cranial AVMs with good tolerance and excellent clinical response. Two patients with chest wall AVMs experienced significant reduction in the volume, redness and deformity of their lesion, alongside a reduction in the overall cardiac output and blood supply to the AVM (81, 82). One of these patients with Cobb syndrome had intermittent drug holidays and observed resurgences of symptoms during these off-periods, which is similar to what is seen in the rapamycin experience in low-flow VMs (83). Importantly, this patient also had a spinal intramedullary component to the AVM that was the first example of a CNS-lesion responding well to MEK inhibition; he had a reduction in shunting over 12 days of daily Trametinib and no serious adverse events (83). The most common side effects of Trametinib include cutaneous rash, gastrointestinal symptoms, fatigue and hair loss/thinning, albeit being mostly mild-to-moderate in severity.

Given the preliminary success of Trametinib for severe AVMs, a new phase II European trial, TRAMAV, has just started to recruit adult patients with severe extracranial AVMs to study the safety and efficacy of Trametinib in this population (EudraCT: 2019-003573-26). Our group is also embarking on a prospective study assessing the safety and efficacy of MEK inhibition for patients in Toronto with palliative extra-cranial and intra-cranial AVMs under compassionate use. Further preclinical work and clinical trials are needed to determine which molecular activities specifically drive AVM pathogenesis and alter its natural history, and then ensure the targeted therapy works on addressing those features in particular with a good tolerance profile. Combining pathway-specific therapeutics (such as MEK inhibitors) with more upstream anti-angiogenic drugs (such as thalidomide) might confer a more synergistic clinical effect while also: (1) reducing drug resistance, and (2) enabling a lower minimal therapeutic dose to mitigate unwanted side effects. These drugs can then act to either bridge a palliative patient to conventional treatments, support conventional treatments such as reducing flow alongside radiosurgery, or potentially facilitating cure in small niduses or residual shunts/recurrences after conventional treatments.

## Minimally invasive techniques in detecting mutational burden

Our understanding of the molecular biology driving VM development has significantly evolved from the first discovery of a somatic mutation causing a venous malformation around a decade ago. With the growing evidence of the various mutational variants in both major cellular signaling pathways and the potential for various systemic therapeutics in VM care, the need for an accurate molecular diagnosis becomes crucial. The early experience with repurposing cancer drugs for VMs involved an exploration of mutational burden using invasive biopsy methods, which was naturally applied to peripheral lesions. Nevertheless, a cutaneous biopsy still holds a risk of bleeding and issues with wound healing; access to deeper lesions and those in the central nervous system pose significant additional safety challenges. To address these concerns, investigators from Italy attempted to recapitulate the mutational burden in a plasma draw, in the form of a modified “liquid biopsy” (84). Liquid biopsies are commonly used in oncology patients where there is a high amount of shedding circulating tumor DNA that can be detected from a peripheral draw and used either for molecular staging or confirmation of response to treatment (85). In AVMs, the amount of cell-free DNA is significantly lower than in tumors, which means that the amount of cell-free DNA in a peripheral draw would be well below the level of detection. Instead, they captured blood from 5 patients that was intimate with the lesion at the time of angiography, alongside paired blood from a peripheral venous draw (negative control). Using next-generation sequencing, they were able to detect known mutations in the isolated cell-free DNA specifically the efferent vein DNA with (1) an allele frequency at an order of magnitude above the limit of detection, and (2) with no serious adverse events from the procedure (84). A subsequent report from Seattle also highlighted the safety and utility of a liquid biopsy technique on plasma derived from 8 peripheral AVMs, 3 VeMs, and cystic fluid samples from 7 LMs; they were able to detect the driving mutation in 25, 33, and 100% of those samples, respectively (86). In one patient with MCAP, a cerebrospinal fluid sample was obtained for cancer staging and a similar method of extracting cell-free DNA from CSF instead of plasma was conducted. *PIK3CA* mutant variants were detected with allele frequency of 3.08%, which is slightly higher than what was reported in the lesional plasma literature, but naturally lower than a cutaneous biopsy (87). Lastly, a slightly more invasive approach was proposed by the group at University of San Francisco termed “endoluminal biopsy” whereby a coil is placed intimate to the wall of the vessel lumen just prior to a planned endovascular treatment session. In doing so, the coil gets coated with genomic mutant cells and upon retrieval, DNA can be isolated for genomic sequencing. They were able to successfully and safely employ this technique to detect mutations including *KRAS* in four patients with brain AVMs (88). These novel minimally-invasive methods in detecting mutational burden in VMs are certainly in their infancy and require further validation so that they can be used safely and in a way that is reliable and accessible, even from very small amounts of DNA.

## Conclusions

A decade's worth of expanding knowledge on the molecular biology of VMs has paved the way for repurposing cancer therapeutics to VM care, with significant promise in the most complex of cases. The ability to detect a patient's unique mutational profile and then safely apply an appropriate cocktail of targeted treatments will be the future of VM precision medicine; further large prospective trials and preclinical work are needed to facilitate our understanding of these complex diseases and expand their treatment potential.

## Author contributions

AM and IR both contributed to the conception of the review, manuscript preparation, and final review. AM conducted literature review and data synthesis. Both authors contributed to the article and approved the submitted version.
